# The Book World of Medicine and Science

**Published:** 1911-07-15

**Authors:** 


### The Book World of Medicine and Science.

Diseases of the Spinal Cord. By R. T. Williamson, M.D., F.R.C.P. Second Impression. (London: Henry Frowde and Hodder and Stoughton. 1911. Price 15s. net.)

It is now two years since Dr. Williamson brought out his treatise on the diseases of the spinal cord, and we hope that the appearance of a second impression may be taken as a sign that encouragement has been accorded to his work. That this really is a good and useful manual on an intricate subject becomes apparent on dipping into its profusely illustrated pages, while a more thorough perusal only serves to confirm the impression that the author is one who has taken infinite pains in his work. To complain that there is but little that is original in the volume is to misconstrue the purpose for which the book was written; this we take to be the compilation of a text-book containing in methodical arrangement all that is worthy of acceptance in the study of the diseases of the spinal cord. For students and general practitioners we know of no book which would be of more practical assistance or from which the fundamental facts of structure and function of the cord and a clear knowledge of its diseases could be more conveniently obtained. The author has been well served by his publishers in the matter of printing, while the illustrations and diagrams alone form a valuable collection which must greatly facilitate the study of the signs and symptoms. The book is divided into some fourteen sections, of which the first four form an admirable compendium of our knowledge of the structure, pathology and functions of the cord and the symptoms of spinal diseases. It is perhaps unavoidable that in a book of this description there should be a certain amount of repetition, but on the other hand there is the advantage that in any particular section one is more likely to get a complete account of the subject. The references to the literature are very numerous, though Dr. Williamson has fortunately restricted the bibliographies at the end of each section to reasonable proportions. As far as his own judgments are concerned, some may be inclined to say that he errs too much on the modest side, but in dealing with the intricacies of the spinal cord this is rather a matter for congratulation, seeing that the author is careful not to obscure the main points but to leave a clear impression of the important features to be memorised by the student. Although the subject-matter was brought fairly well up to date in the first edition, there has now been added a further appendix containing accounts of several of the most important advances which have been made quite recently; notably a reference to work on acute anterior poliomyelitis and tabes dorsalis. Some of the more interesting features of recent epidemics of the former disease are described in this addendum. A useful chapter is the one detailing some of the methods of staining employed for microscopic sections of the cord and their preparation; this will be found of assistance in understanding many of the illustrations. It is a matter of considerable credit that on the first appearance of this manual but few errors were pointed out and these somewhat doubtful ones. The account given of the reflexes is particularly interesting and lucid; especially in regard to the importance of the tendo Achillis jerk and its relation to the knee jerk in tabes, general paralysis, diabetes, and alcoholic heart failure. The question of the state of the reflexes in complete tranverse lesions of the cord is critically considered and the general conclusion arrived at is, that while the rule is for the reflexes to be absent, exceptions do occur. The subject of Lumbar Puncture is, for some reason, dealt with in the chapter on electrical reactions. Though, as we have implied, this volume does not materially advance our knowledge of spinal diseases, it is, none the less, a valuable manual of practical utility, and we have no hesitation in recommending it as a suitable book for students and general practitioners.

A Manual of Gynæcology. By Thomas Watts Eden, M.D., C.M., F.R.C.P. (London: J. and A. Churchill. 1911. Pp. 632. Price 18s. net.)

Following up the success achieved by his "Manual of Midwifery," Dr. Eden has now brought out his long-expected volume on the sister subject of gynæcology. In passing, we may perhaps deplore the fact that it has not been found possible to issue the latter book at the same price as the midwifery manual. As was anticipated, this treatise on gynæcology is a worthy successor to the author's other text-book, and we think it fulfils the promise which his well-known reputation as a teacher of students has held out. In arrangement the book has been planned on a clinical basis rather than on systematic pathology. Considerable care has been taken in explaining fully the anatomy and physiology of the female genital organs and this section is profusely illustrated; indeed, we may venture the criticism that a few of the illustrations are unnecessary, though of their general excellence we must concede a very favourable opinion. The student has been well catered for by Dr. Eden; he must be dull who cannot glean a useful working knowledge of the commoner gynæcological diseases and operations from the illuminating diagrams and the clearly expressed text. It is unnecessary to go into much detail of the subject-matter; suffice it to say that the author has stated well and concisely the current teaching on accepted points, and has, where necessary, on debatable ground, given the best theories in a creditably succinct manner. The inclusion of a description of intermenstrual pain or *mittelschmerz* will undoubtedly be popular in view of the recent question on that subject set at the Conjoint Examination—a question which we believe caused some consternation among the candidates. It is pointed out that *mittelschmerz* is generally associated with some recognisable local lesion, but it cannot in all cases be assumed that the morbid condition discovered in the pelvis is the cause of the pain. Of the causation, that theory is given precedence which suggests that it is due to some abnormality of the function of ovulation. This accounts for its periodic rhythm, for its varying relation to the menstrual period, and for the frequency with which it is associated with inflammatory lesions of the tubes and ovaries. In a book which is remarkably free from examples of unequal treatment of the various divisions of the subject, it is supererogatory to laud any particular sections, but we may mention that in the concluding portion dealing with operative technique we find many excellent illustrations which are very helpful in following the text. We are glad to see that Dr. Eden deprecates the postponement of abdominal operations on account of the appearance of menstruation; a convention which is still unnecessarily given way to. The subject of treatment is in most cases handled in a dogmatic fashion—a method which we believe will be duly appreciated by general practitioners, and one which the practical experience of the author entitles him to use without hesitation. The book is one it is a pleasure to read, and we can confidently recommend it as a highly serviceable text-book and guide for practitioners.

